# Inequalities in pediatric avoidable hospitalizations between Aboriginal and non-Aboriginal children in Australia: a population data linkage study

**DOI:** 10.1186/s12887-016-0706-7

**Published:** 2016-10-21

**Authors:** Kathleen Falster, Emily Banks, Sanja Lujic, Michael Falster, John Lynch, Karen Zwi, Sandra Eades, Alastair H. Leyland, Louisa Jorm

**Affiliations:** 1 National Centre for Epidemiology and Population Health, Australian National University, Canberra, Australia; 2 The Sax Institute, Sydney, Australia; 3 Centre for Big Data Research in Health, University of New South Wales, Sydney, Australia; 4 Centre for Health Research, School of Medicine, University of Western Sydney, Campbelltown, Australia; 5 School of Public Health, University of Adelaide, Adelaide, Australia; 6 Sydney Children’s Hospitals Network, Sydney, Australia; 7 School of Women and Children’s Health, Faculty of Medicine, University of New South Wales, Sydney, Australia; 8 Baker IDI Heart and Diabetes Institute, Melbourne, Australia; 9 MRC/CSO Social and Public Health Sciences Unit, University of Glasgow, Glasgow, Scotland

**Keywords:** Indigenous health, Avoidable hospitalisations, Preventable hospitalisations, Child health, Inequalities

## Abstract

**Background:**

Australian Aboriginal children experience a disproportionate burden of social and health disadvantage. Avoidable hospitalizations present a potentially modifiable health gap that can be targeted and monitored using population data. This study quantifies inequalities in pediatric avoidable hospitalizations between Australian Aboriginal and non-Aboriginal children.

**Methods:**

This statewide population-based cohort study included 1 121 440 children born in New South Wales, Australia, between 1 July 2000 and 31 December 2012, including 35 609 Aboriginal children. Using linked hospital data from 1 July 2000 to 31 December 2013, we identified pediatric avoidable, ambulatory care sensitive and non-avoidable hospitalization rates for Aboriginal and non-Aboriginal children. Absolute and relative inequalities between Aboriginal and non-Aboriginal children were measured as rate differences and rate ratios, respectively. Individual-level covariates included age, sex, low birth weight and/or prematurity, and private health insurance/patient status. Area-level covariates included remoteness of residence and area socioeconomic disadvantage.

**Results:**

There were 365 386 potentially avoidable hospitalizations observed over the study period, most commonly for respiratory and infectious conditions; Aboriginal children were admitted more frequently for all conditions. Avoidable hospitalization rates were 90.1/1000 person-years (95 % CI, 88.9–91.4) in Aboriginal children and 44.9/1000 person-years (44.8–45.1) in non-Aboriginal children (age and sex adjusted rate ratio = 1.7 (1.7–1.7)). Rate differences and rate ratios declined with age from 94/1000 person-years and 1.9, respectively, for children aged <2 years to 5/1000 person-years and 1.8, respectively, for ages 12- < 14 years. Findings were similar for the subset of ambulatory care sensitive hospitalizations, but in contrast, non-avoidable hospitalization rates were almost identical in Aboriginal (10.1/1000 person-years, (9.6–10.5)) and non-Aboriginal children (9.6/1000 person-years (9.6–9.7)).

**Conclusions:**

We observed substantial inequalities in avoidable hospitalizations between Aboriginal and non-Aboriginal children regardless of where they lived, particularly among young children. Policy measures that reduce inequities in the circumstances in which children grow and develop, and improved access to early intervention in primary care, have potential to narrow this gap.

**Electronic supplementary material:**

The online version of this article (doi:10.1186/s12887-016-0706-7) contains supplementary material, which is available to authorized users.

## Background

It is well established that Australian Aboriginal children start life with a disproportionate burden of social and health disadvantage [1–3]. The early life disadvantage experienced by Aboriginal children is a precursor to adverse outcomes later in life. Aboriginal Australians experience worse health, development, education and employment outcomes than non-Aboriginal Australians through child- and adult-hood [1, 2, 4–7]. There is an identified need for better evidence for targeting and evaluating the impact of policy, programs and services on closing modifiable health gaps during early childhood [8].

Routinely collected population data can provide unique insights into the magnitude and nature of health problems affecting large numbers of people, as well as making visible the experience of smaller sub-populations. Rates of avoidable hospitalization were originally conceived as an indicator of access to quality out-of-hospital care [9]. These indicators use routinely collected hospital data and usually include a set of diagnosis and procedure codes for conditions that are considered amendable to non-hospital interventions. Like many countries, Australian government agencies routinely report on avoidable hospitalizations for a range of acute, chronic and vaccine-preventable conditions [10]. However, when it comes to children, the Australian indicator may have limited relevance because it includes a number of predominantly adult diseases [10].

The United States Agency for Healthcare Research and Quality first published a set of ‘Pediatric Quality Indicators’ to identify hospitalizations in children that may be avoidable via changes at the health system or provider level in 2006 [11]. More recently, a pediatric avoidable hospitalization indicator was developed in New Zealand that reframed the concept of ‘avoidable’ to include conditions that might be influenced not only by primary care, but also broader policy measures, such as provision of affordable and quality housing, childcare and income support [12]. This broader definition of avoidable hospitalizations is useful because it avoids potentially unfair and unrealistic expectations about the extent to which reductions in hospitalizations might be achieved through primary care alone [12].

To our knowledge, Australian children have been included in few studies of avoidable hospitalizations to date [13–17], and only two have provided a breakdown by Aboriginal status [16, 17]. A study of aggregate hospital separation data from five de-identified Australian states and territories in 1993–94 reported higher rates of hospitalization for select child-relevant ambulatory care sensitive conditions in Aboriginal compared with non-Aboriginal children [17]. Aboriginal 0–14 year old children were also found to have higher avoidable hospitalization rates than their non-Aboriginal peers in the Northern Territory in 1998–2006, although a pediatric indicator was not used [16]. Moreover, rates were mostly reported for broad age groups, which is problematic because pediatric avoidable hospitalization rates are highest in the first two years of life [18]. Because of the identified need to target and monitor modifiable health gaps between Australian Aboriginal and non-Aboriginal children, we aim to quantify inequalities in pediatric avoidable hospitalizations between Aboriginal and non-Aboriginal children in the most populous state of Australia, New South Wales (NSW), by applying a pediatric avoidable hospitalization indicator to linked hospital data for children born between July 2000 and December 2012.

## Methods

### Data sources

We used hospital data from the NSW Admitted Patients Data Collection, which includes records of all separations from public and private hospitals and day procedure centers in NSW. Each record represents an episode of care that ends when a patient is transferred to another type of care, discharged from hospital, or dies. Patient demographics and multiple diagnoses and procedures are recorded for each separation. Diagnoses are coded according to the Australian modification of the International Statistical Classification of Diseases and Related Problems 10th Revision (ICD-10-AM) [19] and procedures according to the Australian Classification of Health Interventions [19]. We also used death registration data from the NSW Register of Births, Deaths and Marriages to ascertain children who died during the study period. Approval to link and use these data was obtained from the relevant data custodians (NSW Ministry of Health and NSW Register of Births, Deaths and Marriages) prior to seeking ethical approval.

Data were linked by the NSW Centre for Health Record Linkage using probabilistic methods that match identifiers common to the records being linked (e.g. name, sex, date of birth, address) [20]. Only a de-identified unique project person number and information about hospitalizations and/or deaths that occurred between 1 July 2000 and 31 December 2013 were released to the researchers.

### Setting

NSW is Australia’s most populous state. The 2006 Australian Census, which is the approximate mid-point for this study period, estimated approximately 6.8 million residents in NSW, including almost 150 000 (2.2 %) Aboriginal and/or Torres Strait Islander people [21]. Henceforth, we refer to Aboriginal and/or Torres Strait Islander people as ‘Aboriginal’ because Torres Strait Islander people accounted for 0.1 % of the NSW population in 2006 [21]. In 2006, 73 % of the NSW population lived in a major city, 27 % lived in regional areas, and less than 1 % lived in remote areas [21]. In contrast, 43 % of the NSW Aboriginal population lived in a major city in 2006, 52 % lived in regional areas, and 5 % lived in remote areas [21].

Australia’s universal public health insurance scheme (Medicare) covers the cost of necessary health care to individuals admitted as public patients in public hospitals [22]. The Medicare Benefits Schedule sets fees for medical services provided in primary care settings; however, there is variation in the amount and method in which patients are charged for these services. General practitioners (GPs) can directly bill Medicare for services provided (known as ‘bulk billing’), in which case the patient incurs no cost. GPs also have the option to charge the patient the Medicare Benefits Schedule fee, and the patient may seek reimbursement from Medicare. The GP is also entitled to charge more than the Medicare Benefits Schedule fee, in which case the patient incurs the cost of the ‘gap’ between the charged amount and the Medicare Benefits Schedule fee.

### Study population

Children and their records were included in this analysis if they were born in a NSW hospital between 1 July 2000 and 31 December 2012, and their area of residence was within the state of NSW (*n* = 1 124 717). We defined birth admissions as hospital records with a ‘live born infant’ ICD-10-AM diagnosis code (i.e. Z38) or a date of birth within the hospital admission and separation dates. From this group, 3277 children were excluded because: their sex was coded as indeterminate or missing (*n* = 34); there were discrepancies in their date of birth, admission and/or separation date on their birth record (*n* = 289); or they died before 29 days of age (*n* = 2954). A total of 1 121 440 children were included in this analysis (Table 1).

**Table 1:** Characteristics and person years of follow-up time (2000–2013) for Aboriginal and non-Aboriginal children in a population cohort born between July 2000 and December 2012 in New South Wales, Australia

	Non-Aboriginal			Aboriginal		
	*N*	%	Person years	*N*	%	Person years
Total	1,085,831	100	7,681,406	35,609	100	223,190
Birth year						
Jul 2000–Dec 2004	367,542	34	4,125,434	9,047	25	100,296
Jan 2005–Dec 2008	353,945	33	2,466,933	11,607	33	79,332
Jan 2009–Dec 2012	364,344	34	1,089,040	14,955	42	43,562
Contribution of follow-up to age group^a^						
Less than 2 years	-	-	2,123,515	-	-	68,971
2–4 years	-	-	1,802,906	-	-	55,189
4–6 years	-	-	1,440,318	-	-	41,123
6–8 years	-	-	1,081,702	-	-	28,451
8–10 years	-	-	736,009	-	-	18,066
10–12 years	-	-	406,279	-	-	9,431
12–13 years	-	-	90,678	-	-	1,959
Sex						
Female	527,500	49	3,729,845	17,251	48	108,591
Male	558,331	51	3,951,561	18,358	52	114,599
Low birth weight and/or premature birth						
No	1,017,508	94	7,212,029	32,039	90	201,599
Yes	68,323	6	469,377	3,570	10	21,591
Private patient and/or health insurance						
No	702,362	65	5,049,008	34,531	97	217,477
Yes	383,469	35	2,632,399	1,078	3	5,713
Geographical remoteness						
Major city	730,967	67	5,105,713	10,238	29	64,754
Inner regional	262,887	24	1,899,768	13,231	37	79,583
Outer regional	84,425	8	617,719	8,837	25	56,053
Remote/Very remote	7,552	1	58,205	3,303	9	22,800
Area-level socio-economic disadvantage^b^						
First quintile (Most disadvantaged)	199,716	18	1,443,310	16,719	47	107,615
Second quintile	235,069	22	1,669,550	9,746	27	59,739
Third quintile	219,196	20	1,560,849	6,210	17	37,857
Fourth quintile	225,527	21	1,569,176	2,344	7	14,415
Fifth quintile (Least disadvantaged)	206,323	19	1,438,522	590	2	3,564

^a^Children contribute person years of follow-up from the date they are 29 days old until their death or the end of the study period (December 31, 2013). Many of the children contribute person years of follow-up to more than one age group during the study period; ^b^Socio-economic indices for Areas (SEIFA) Index of Relative Socio-Economic Advantage and Disadvantage based on the child’s statistical local area of residence at birth

### Analysis variables

Our main outcome was pediatric potentially avoidable hospitalizations, as defined by Andersen et al. [12]. We also report ambulatory care sensitive and non-avoidable hospitalizations [12]. We used the primary diagnosis to identify avoidable, ambulatory care sensitive or non-avoidable hospitalizations (Additional file 1: Table S1). Admissions occurring before children were 29 days old were excluded. Hospitalizations for vaccine preventable diseases were classified as avoidable if the child’s age was greater than or equal to the recommended immunisation age for each condition in NSW [23]. Admissions for which the emergency status was coded as ‘planned’ were excluded from the count of avoidable hospitalizations, except for dental conditions, because most planned admissions are unlikely to be avoidable.

The following individual- and area-level covariates were in the hospital data: Aboriginality; age; sex; low birth weight (<2500 g) and/or prematurity (<37 weeks gestation); private health insurance/patient status; remoteness of residence, measured by the Accessibility/Remoteness Index of Australia Plus (ARIA+) [24]; and area socioeconomic disadvantage, measured by the Australian Bureau of Statistics Socioeconomic Indexes for Areas (SEIFA) [25]. The child’s Aboriginality recorded in the hospital data is based on the response to the question ‘Is this child of Aboriginal or Torres Strait Islander origin?’, which is asked directly of the mother at birth, and of a parent or guardian at admission for children aged less than 15 years. Although the number of Aboriginal people identified in hospital data increases with the use of multiple record algorithms [26–28], we assigned Aboriginality from the birth record to avoid introducing misclassification bias whereby more frequently hospitalized children have more opportunity to be recorded as Aboriginal, either correctly or in error. For the same reason, we assigned other variables from the birth admission. We calculated the child’s age on admission as the difference between their dates of birth and admission.

### Data analysis

We calculated person-years of follow-up for each child from the date they were 29 days old until 31 December 2013, or their date of death. We estimated admission rates (ARs) per 1000 person-years by dividing the number of admissions by the person-years accumulated, and multiplying by 1000. We calculated 95 % confidence intervals (CIs) assuming a Poisson distribution of events. Rate differences were calculated by subtracting the AR for non-Aboriginal children from the AR for Aboriginal children. Aboriginal to non-Aboriginal admission rate ratios (ARRs) for each outcome and age group were calculated by dividing the Aboriginal AR by the non-Aboriginal AR. We calculated the proportion of avoidable hospital admissions that occurred outside of standard general medical practice hours (i.e. 08:00–18:00) [29].

To account for differences in age and sex, negative binomial models were used to estimate adjusted ARRs for Aboriginal to non-Aboriginal children for each outcome and condition, modeling the number of hospitalizations as an outcome (using a log link), and including terms for age (in two year age groups), sex and Aboriginal status, with the log of the person-years of follow-up as an offset (Model 1). To account for clustering within geographic statistical local areas (henceforth, ‘areas’), a random intercept term was added to the model, which allowed the baseline admission rate to vary between areas, creating a multi-level model (Model 2). In the multilevel model, we then explored whether any of the inequality in study outcomes reflected differences between Aboriginal and non-Aboriginal children in terms of other measured covariates (Model 3). To test whether the effect of covariates on each outcome were the same for Aboriginal and non-Aboriginal children, we tested interaction terms between Aboriginal status and each covariate in multilevel models adjusted for age, sex, and variation in rates between areas (by including a random intercept term for areas).

We used SAS 9.3 [30], MLwiN 2.25 [31], and R 2.15.0 for analyses [32]. Multilevel modeling in MLwiN used generalized least squares (IGLS) estimation and a 2nd order PQL approximation.

## Results

We identified 365 386 potentially avoidable hospitalizations among the 1 121 440 children born between 1 July 2000 and 31 December 2012, who were followed from birth until 31 December 2013 (Additional file 1: Table S2). Of these, 243 643 hospitalizations were considered ambulatory care sensitive. The avoidable hospitalization rate was 90.1 per 1000 person-years (95 % CI, 88.9–91.4) in Aboriginal children compared with 44.9 per 1000 person-years (95 % CI, 44.8–45.1) in non-Aboriginal children (Additional file 1: Table S2). The ambulatory care sensitive hospitalization rate was 56.7 (95 % CI, 55.7–57.7) and 30.1 (95 % CI, 29.9–30.2) per 1000 person-years in Aboriginal and non-Aboriginal children, respectively. Of the ambulatory care sensitive hospitalizations, 56 % and 57 % of admissions occurred outside standard general practice hours for Aboriginal and non-Aboriginal children, respectively. The non-avoidable hospitalization rate was 10.1 (95 % CI, 9.6–10.5) and 9.6 (95 % CI, 9.6–9.7) per 1000 person years in Aboriginal and non-Aboriginal children.

**Fig. 1:**
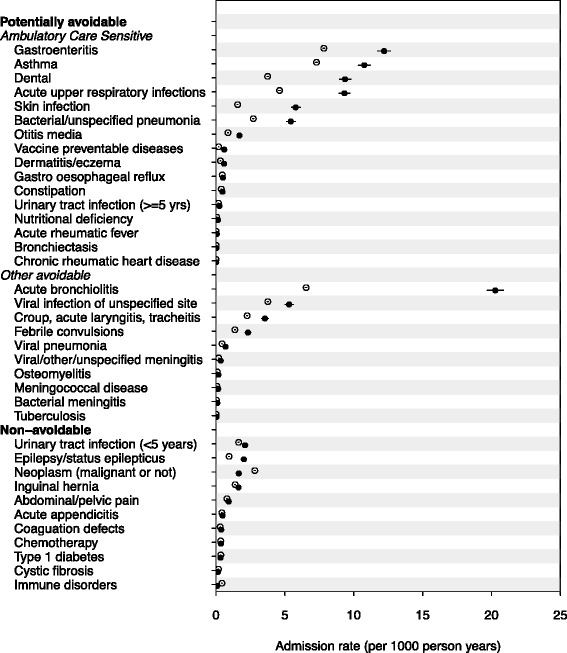
Title: Admission rates for potentially avoidable and non-avoidable hospitalizations (2000–2013) in a population cohort of Aboriginal and non-Aboriginal children born 2000–2012, New South Wales, Australia. Legend: closed circles, Aboriginal; open circles, non-Aboriginal; Error bars (through circles) are 95 % confidence intervals. Sorted by Aboriginal admission rates in descending order. Admission rates, rate differences, rate ratios and 95 % confidence intervals are shown in Additional file 1: Table S2

The five most common causes of avoidable hospitalization among Aboriginal children in the cohort were acute bronchiolitis (AR, 20.3; 95 % CI, 19.7–20.9), gastroenteritis (AR, 12.2; 95 % CI, 11.7–12.7), asthma (AR, 10.8; 95 % CI, 10.3–11.2), dental conditions (AR, 9.4; 95 % CI, 9.0–9.8), and acute upper respiratory tract infections (URTIs) (AR, 9.3; 8.9–9.7) (Fig. 1). Four of the five most common causes were the same for non-Aboriginal children, but with lower rates: gastroenteritis (AR, 7.8; 95 % CI, 7.8–7.9), asthma (AR, 7.3; 95 % CI, 7.2–7.4), acute bronchiolitis (AR, 6.5; 95 % CI, 6.5–6.6), acute URTIs (AR, 4.6; 95 % CI 4.6–4.7) and viral infection of unspecified site (AR, 3.8; 95 % CI 3.7–3.8) (Fig. 1). The conditions with the greatest Aboriginal to non-Aboriginal rate difference were acute bronchiolitis (RD, 13.8), dental (RD, 5.7), acute URTIs (RD, 4.7), gastroenteritis (RD, 4.4) and skin infections (4.2) (Additional file 1: Table S2).

**Fig. 2:**
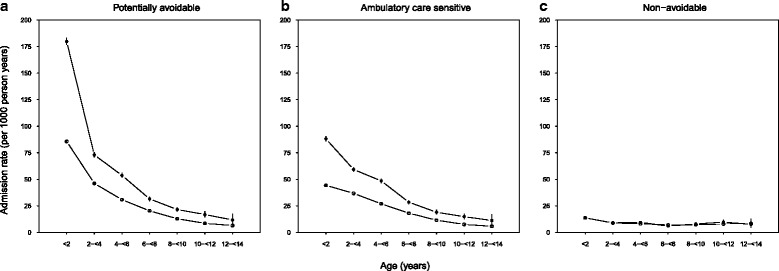
Title: Age-specific admission rates for potentially avoidable, ambulatory care sensitive and non-avoidable hospitalizations (2000–2013) in a population cohort of Aboriginal and non-Aboriginal children born 2000–2012, New South Wales, Australia. Legend: closed circles, Aboriginal; open circles, non-Aboriginal. Error bars (through circles) are 95 % confidence intervals

For Aboriginal and non-Aboriginal children, avoidable hospitalization rates were highest among those aged less than two years (179.9 and 85.7 per 1000 person-years, respectively) and decreased to less than 10 per 1000 person-years among children aged 12 to <14 years (Fig. 2). Among children less than two years of age, Aboriginal children were 1.9 times more likely to be admitted for an avoidable hospitalization than non-Aboriginal children, and the rate difference was 94.1 per 1000 person-years (Fig. 2, Table 2). The rate difference between Aboriginal and non-Aboriginal children declined to 5.3 per 1000 person-years by 12 to <14 years of age.

**Table 2:** Age-specific Aboriginal to non-Aboriginal rate differences and rate ratios for potentially avoidable, ambulatory care sensitive and non-avoidable hospitalisations (2000–2013) in a population cohort of Aboriginal and non-Aboriginal children born between July 2000 and December 2012 in New South Wales, Australia

Age group	Potentially avoidable			Ambulatory care sensitive			Non-avoidable		
	Rate difference (RD)	Rate ratio (RR) (95 % CI)		RD	RR (95 % CI)		RD	RR (95 % CI)	
0–< 2 years	94.1	1.9	(1.8–1.9)	43.79	1.9	(1.8–1.9)	- < 0.1	0.8	(0.7–0.9)
2–< 4 years	26.8	1.5	(1.5–1.6)	22.49	1.6	(1.5–1.6)	<0.1	0.8	(0.7–0.9)
4–< 6 years	22.9	1.7	(1.6–1.8)	21.58	1.8	(1.7–1.9)	1.2	1.0	(0.7–1.4)
6–< 8 years	11.3	1.5	(1.4–1.6)	10.31	1.6	(1.4–1.7)	−1.1	0.9	(0.6–1.2)
8–< 10 years	8.7	1.7	(1.5–1.9)	7.45	1.6	(1.5–1.8)	0.7	1.1	(0.9–1.3)
10–< 12 years	8.3	2.0	(1.7–2.3)	7.32	2.0	(1.6–2.3)	1.7	1.2	(0.9–1.6)
12–< 14 years	5.3	1.8	(1.2–2.8)	5.46	2.0	(1.3–3.0)	–0.7	1.0	(0.6–1.6)

*CI* confidence interval, *RD* rate difference, *RR* rate ratio

**Fig. 3:**
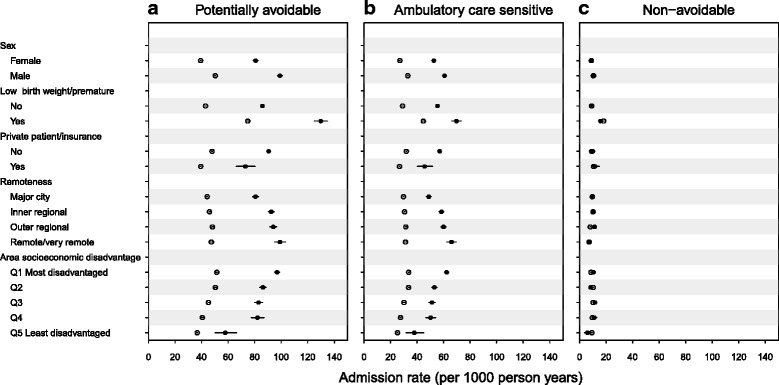
Title: Admission rates for (**a**) potentially avoidable, (**b**) ambulatory care sensitive and (**c**) non-avoidable hospitalizations (2000–2013) by individual- and area-level characteristics in a population cohort of Aboriginal and non-Aboriginal children born 2000–2012, New South Wales, Australia. Legend: closed circles, Aboriginal; open circles, non-Aboriginal. Error bars (through circles) are 95 % confidence intervals

**Fig. 4:**
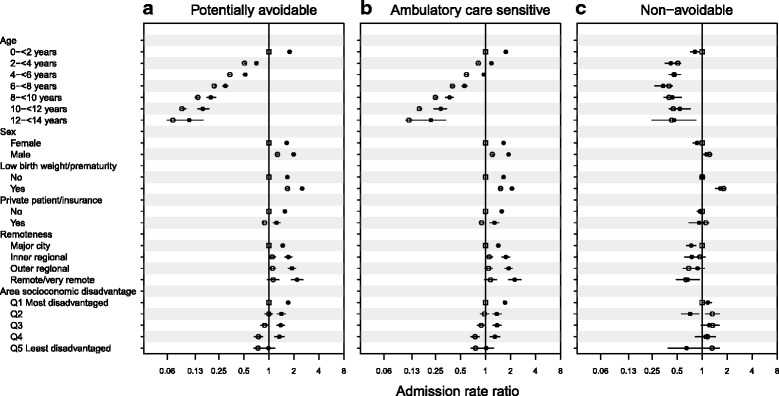
Title: Admission rate ratios for (**a**) potentially avoidable, (**b**) ambulatory care sensitive, and (**c**) non-avoidable hospitalizations for interactions between individual- and area-level characteristics and Aboriginal status from multilevel negative binomial models with a random intercept for Statistical Local Area, adjusted for age and sex. Rate ratios are relative to the reference group. Legend: closed circles, Aboriginal; open circles, non-Aboriginal; open square, non-Aboriginal reference group. Error bars (through symbols) are 95 % confidence intervals

Aboriginal children had higher avoidable hospitalization rates across all categories of sex, low birth weight/prematurity, private health insurance/patient status and remoteness or disadvantage of the area where children started life (Fig. 3). In particular, the relative difference in avoidable hospitalizations between those living in remote areas versus major cities was greater for Aboriginal versus non-Aboriginal children; compared with non-Aboriginal children living in major cities (ARR, 1.0; reference group), Aboriginal children living in remote areas and major cities were 2.2 (95 % CI, 1.9–2.6) and 1.5 (95 % CI, 1.4–1.5) times more likely to be admitted for an avoidable hospitalization, respectively (Fig. 4). In contrast, non-Aboriginal children living in remote areas were 1.1 (95 % CI, 1.0–1.3) times more likely to be admitted for an avoidable hospitalization than non-Aboriginal children living in major cities.

After adjusting for differences in age and sex (Model 1), Aboriginal children were 1.7 times as likely to have an avoidable hospitalization as non-Aboriginal children in the cohort (Table 3). When variation in admission rates between areas was also accounted for (Model 2), the Aboriginal to non-Aboriginal admission rate ratio for avoidable hospitalizations was 1.6 (95 % CI, 1.6–1.6). After accounting for differences in individual- and area-level characteristics (Model 3), the Aboriginal to non-Aboriginal admission rate ratio was 1.6 (95 % CI, 1.6–1.7). The magnitude of the inequality was similar for the subset of ambulatory care sensitive hospitalizations, after adjustment for age and sex (ARR, 1.7; 95 % CI, 1.7–1.8), and after inclusion of random intercept and adjustment for all covariates (ARR,1.6; 95 % CI, 1.6–1.6) (Table 3). In contrast, Aboriginal children were marginally less likely to be admitted for a non-avoidable hospitalization than non-Aboriginal children (Table 3; ARR, 0.9; 95 % C, 0.8–1.0) after adjustment for age, sex and variation in admission rates between areas.

**Table 3:** Aboriginal to non-Aboriginal admission rate ratios from multilevel models for potentially avoidable, ambulatory care sensitive and non-avoidable hospitalisations (July 2000 to December 2013) in a population cohort of children born between July 2000 and December 2012 in New South Wales, Australia

Model	Variables and random effects added to the model:	Aboriginal to non-Aboriginal Rate Ratio	95 % confidence interval	
Potentially avoidable hospitalisations				
1	Age and sex	1.73	1.71	1.75
2	Model 1 + random intercept for area	1.60	1.58	1.62
3	Model 2 + individual^a^- and area^b^-level characteristics	1.60	1.58	1.61
Ambulatory care sensitive hospitalisations				
1	Age and sex	1.74	1.72	1.77
2	Model 1 + random intercept for area	1.60	1.58	1.62
3	Model 2 + individual^a^- and area^b^-level characteristics	1.57	1.55	1.59
Non-avoidable hospitalisations				
1	Age and sex	0.89	0.81	0.97
2	Model 1 + random intercept for area	0.90	0.82	0.97
3	Model 2 + individual^a^- and area^b^-level characteristics	1.03	0.96	1.10

^a^Low birth weight/prematurity and private health insurance/patient status; ^b^geographical remoteness and area socio-economic disadvantage

## Discussion

We found that avoidable hospitalization rates were almost double in Aboriginal compared with non-Aboriginal children less than two years of age, and the absolute difference in rates was 94 per 1000 person-years. Although the absolute and relative inequalities were present across all ages, the absolute difference in rates between Aboriginal and non-Aboriginal children declined to 5 per 1000 person-years by 12 to <14 years of age. Respiratory and infectious conditions were the most common reasons for avoidable hospitalizations in all children, although Aboriginal children were admitted more frequently for all conditions. We also found that the impact of living in more remote and disadvantaged areas on a child’s risk of avoidable hospitalization was greater for Aboriginal children.

To our knowledge, this is the first Australian study to reveal that avoidable hospitalizations are highest in children in the first two years of life and decrease among older children, consistent with findings in New Zealand [18]. For the first time, we demonstrated that absolute differences in avoidable hospitalizations between Aboriginal and non-Aboriginal children were greatest in children less than two years, while the magnitude of the relative inequality was similar across age groups. In most contexts, both the absolute and relative differences between two groups matter [33]. That the relative inequality is similar across age groups suggests there is a general problem that requires a systemic approach. On the other hand, the greater absolute differences in avoidable hospitalizations between Aboriginal and non-Aboriginal children less than two years of age indicate there may be scope to reduce avoidable hospitalizations via targeted prevention and early intervention measures that reduce disease burden and improve access to treatment for common childhood conditions.

Respiratory and infectious conditions were the most common reasons for avoidable hospitalization among all children in the cohort, with Aboriginal children more likely to be admitted for all conditions. These findings are consistent with other Australian data on common conditions resulting in pediatric emergency department presentations [34] and higher hospitalization rates for respiratory diseases [35–39] and gastroenteritis [38, 40–42] among Aboriginal children in Western Australia. Although we were unable to ascertain the burden of these conditions outside of the hospital setting, other studies have previously reported a high burden of respiratory diseases [43–46], skin infections [47] and otitis media [48–50] in Aboriginal children, particularly in remote communities. Moreover, some important exposures associated with respiratory and infectious conditions, such as smoking [51, 52] and poor housing conditions [53], are known to be common in Aboriginal families [54–56] and associated with poverty. Dental conditions were also a common cause of avoidable hospitalization for Aboriginal children. Previous studies have also documented poor dental health in Aboriginal communities [57–59], as well as higher rates of hospitalization for dental conditions among Aboriginal children in Western Australia [60, 61].

The remoteness or disadvantage of the area where a child lives has previously been associated with avoidable hospitalizations in children in the Australian state of Victoria [13, 14] and New Zealand [18]. What this study shows is that living in more remote or disadvantaged areas has a greater impact on a child’s risk of avoidable hospitalization if they are Aboriginal. Aboriginal families living in remote and disadvantaged areas experience a disproportionate burden of the determinants of poor child health (e.g. overcrowded housing [54]). Barriers to accessing primary care for Aboriginal families – including some that may disproportionately impact on those living in remote and disadvantaged areas – have previously been identified, including: the physical availability of health services (which are clustered in major cities and more advantaged areas), transport, flexible service delivery, affordability, and the cultural acceptability and appropriateness of health services [62]. Health literacy, physician behaviour and hospital admission practices may also impact on whether a child is admitted to hospital.

Because Australia’s universal health insurance scheme (Medicare) covers the cost of necessary health care provided to public patients admitted to public hospitals [22], costs associated with hospitalisation should not have been a determinant of rates of avoidable hospitalisation in this study. However, families may incur costs for seeking GP care because Australian GPs have the option to charge patients more than the Medicare Benefits Schedule fee. During the study period, NSW patients paid an out-of-pocket ‘gap’ payment for 14–24 % of all GP visits [63]. Therefore, costs associated with GP services may deter some families from seeking primary health care for their children in a timely manner.

Use of population data in this study conferred the advantage of a large cohort; this enabled us to reliably quantify the magnitude of the inequality in avoidable hospitalizations between Aboriginal and non-Aboriginal children in narrow age bands for the first time, which is important for guiding policy. Importantly, it has also made visible the health experience of Aboriginal children, a small and disadvantaged sub-population. Other strengths of the study include the length of follow-up for children born earlier in the study period and minimal loss to follow-up (children account for a small proportion of the 3 % of people who migrate outside of NSW each year [64, 65]).

Potential limitations of the outcome measure must be considered. The extent to which the avoidable hospitalizations identified in this study were truly avoidable is unknown. Recent research suggests that socio-demographic and health factors explain more of the geographic variation in adult avoidable hospitalizations than general practitioner supply [66], and our main outcome measure was not focused on primary care as the sole strategy for reducing avoidable hospitalizations. However, we found similar inequalities for avoidable hospitalizations and the subset of ambulatory care sensitive hospitalizations. Despite living in the same area, Aboriginal and non-Aboriginal children may not realise equal access to available primary care services, for reasons including transport difficulties and cultural barriers [62]. In this study, more than half the ambulatory care sensitive hospitalizations occurred outside office hours. From our data, we were unable to determine whether these admissions were for severe and rapid onset illness that would have resulted in hospitalization under any circumstances, or whether improved access to primary care in the days and hours prior to hospitalization might have prevented some of these hospitalizations.

A limitation of using routinely collected hospital data is the under-recording of Aboriginal status [26, 67–69]. These errors are not randomly distributed across hospitals and areas; past audits have shown Aboriginal status has been recorded more accurately in remote areas and that recording of Aboriginal status has improved over time [68, 69]. We aimed to minimize bias introduced in this study by deriving both the numerator and the denominator from the hospital data, rather than deriving the denominator from census data. Another shortcoming is the limited set of covariates available in the data. Private health insurance/patient status was the only individual-level indicator of socioeconomic advantage in the data, and only 3 % of Aboriginal children had private health insurance or were admitted as a private patient compared with 35 % of non-Aboriginal children. The higher proportion of Aboriginal children with low birth weight and/or prematurity in this cohort likely reflects a greater burden of socioeconomic disadvantage among Aboriginal children, but measures such as household income and parent education level were not available. As such, our modeling shows the effect of Aboriginality combined with other unmeasured covariates.

## Conclusions

In an equitable world, there should be no difference in avoidable hospitalizations between Aboriginal and non-Aboriginal children. We observed substantial inequalities in these hospitalizations between Aboriginal and non-Aboriginal children regardless of where they lived, but particularly among very young children. An important question is: How can we close this gap? Broad policy measures that aim to reduce inequities in the circumstances in which children grow and develop (e.g. better quality and affordable housing) may impact on the incidence of common childhood conditions in Aboriginal children, including hospitalization for these conditions [12, 70]. Increased access to early intervention in primary care, particularly for young Aboriginal children, and those living in remote and disadvantaged areas, may also impact on avoidable hospitalizations. Finally, this study provides a novel source of baseline population data to inform the future impact of policies and interventions on existing inequalities.

## Additional file

Additional file 1: Table S1. (ICD-10-AM codes) and **Table S2.** (Potentially avoidable, ambulatory care sensitive and non-avoidable hospitalisation admission rates). **Table S1.** title “List of ICD-10-AM codes used to identify potentially avoidable, ambulatory care sensitive and non-avoidable hospitalisations (adapted from Andersen et al., 2012)”, and **Table S2.** title “Potentially avoidable, ambulatory care sensitive and non-avoidable hospitalisation admission rates (2000–2013) in a population cohort of Aboriginal and non-Aboriginal children born between July 2000 and December 2012 in New South Wales, Australia”. (DOCX 61 kb)

## Abbreviations

ARRs: Admission rate ratios
ARs: Admission rates
CIs: Confidence intervals
ICD-10-AM: International Statistical Classification of Diseases and Related Problems 10th Revision
NSW: New South Wales
