# Supplementary material for: Inequalities in pediatric avoidable hospitalizations between Aboriginal and non-Aboriginal children in Australia: a population data linkage study
Source: BMC Pediatr. 2016 Oct 21;16:169. doi: 10.1186/s12887-016-0706-7 (PMC5073450; doi:10.1186/s12887-016-0706-7)
Supplement: Additional file 1: Table S1. — (ICD-10-AM codes) and Table S2. (Potentially avoidable, ambulatory care sensitive and non-avoidable hospitalisation admission rates). Table S1. title “List of ICD-10-AM codes used to identify potentially avoidable, ambulatory care sensitive and non-avoidable hospitalisations (adapted from Andersen et al., 2012)”, and Table S2. title “Potentially avoidable, ambulatory care sensitive and non-avoidable hospitalisation admission rates (2000–2013) in a population cohort of Aboriginal and non-Aboriginal children born between July 2000 and December 2012 in New South Wales, Australia”. (DOCX 61 kb) [file 12887_2016_706_MOESM1_ESM.docx]

# Additional file 1

**Table S1** List of ICD-10-AM codes used to identify potentially avoidable, ambulatory care sensitive and non-avoidable hospitalisations (adapted from Andersen et al, 2012).

**Table S2** Potentially avoidable, ambulatory care sensitive and non-avoidable hospitalisation admission rates (2000-2013) in a population cohort of Aboriginal and non-Aboriginal children born between July 2000 and December 2012 in New South Wales, Australia.

Table S1. List of ICD-10-AM codes used to identify potentially avoidable, ambulatory care sensitive and non-avoidable hospitalisations (adapted from Andersen et al, 2012).

| **Condition** | **ICD-10-AM codes** |
| --- | --- |
| ***Potential avoidable hospitalisations*** |  |
| *Ambulatory care sensitive hospitalisations* |  |
| Acute rheumatic fever | I00-I02 |
| Acute upper respiratory tract infection excluding croup | J00-J03, J06 |
| Asthma | J45, J46 |
| Bacterial/unspecified pneumonia | J13-J16, J18 |
| Bronchiectasis | J47 |
| Chronic rheumatic heart disease | I05-I09 |
| Constipation | K59.0 |
| Dental (dental caries, pulp, periodontal) | K02, K04, K05 |
| Dermatitis/eczema | L20-L30 |
| Gastro oesophageal reflux | K21 |
| Gastroenteritis | A00-A09, R11, K52.9 |
| Nutritional deficiency | E40-E64, D50-D53 |
| Otitis media | H65-H67 |
| Skin infection | L00-L05, L08, L98.0, J34.0, H01.0, H00.0 |
| Urinary tract infection (≥5 years of age) | N10, N12, N13.6, N30.0, N30.9, N39.0 |
| Vaccine preventable diseases | A33-A37, A80, B05, B06, B16, B18.0, B18.1, B26, M01.4, P35.0 |
| *Other avoidable hospitalisations* |  |
| Acute bronchiolitis | J21 |
| Bacterial meningitis | G00, G01 |
| Croup, acute laryngitis, tracheitis | J04, J05.0 |
| Febrile convulsions | R56.0 |
| Meningococcal disease | A39 |
| Osteomyelitis | M86 |
| Tuberculosis | A15-A19 |
| Viral infection of unspecified site | B34 |
| Viral pneumonia | J12, J10.0, J11.0 |
| Viral/other/unspecified meningitis | A87, G02, G03 |
| ***Non-avoidable hospitalisations*** |  |
| Abdominal/pelvic pain | R10 |
| Acute appendicitis | K35 |
| Chemotherapy | Z51.1 |
| Coagulation defects | D65-D69 |
| Cystic fibrosis | E84 |
| Epilepsy/status epilepticus | G40, G41 |
| Immune disorders | D80-D89 |
| Inguinal hernia | K40 |
| Neoplasm (malignant or not) | C00-D48 |
| Type 1 diabetes | E10 |
| Urinary tract infection (<5 years of age) | N10, N12, N13.6, N30.0, N30.9, N39.0 |

Table S2. Potentially avoidable, ambulatory care sensitive and non-avoidable hospitalisation admission rates (2000-2013) in a population cohort of Aboriginal and non-Aboriginal children born between July 2000 and December 2012 in New South Wales, Australia.

|  | **Non-Aboriginal**  (Total person-years follow-up = 7 681 406) | | | **Aboriginal**  (Total person-years follow-up = 223 190) | | | **RD** | **ARR** | **aARR****^c^**  **(95% CI)** | |
| --- | --- | --- | --- | --- | --- | --- | --- | --- | --- | --- |
|  | **Admissions**  **(N)** | **Admission Rate (AR)****^a^** | **95% CI** | **Admissions**  **(N)** | **AR ^a^** | **95% CI** |  |  |  |  |
| **Potentially avoidable hospitalisations** | 345 274 | 44.9 | (44.8 - 45.1) | 20 112 | 90.1 | (88.9 - 91.4) | 45.2 | 2.0 | 1.7 | (1.7 - 1.7) |
| *Ambulatory care sensitive hospitalisations* | 230 983 | 30.1 | (29.9 - 30.2) | 12 660 | 56.7 | (55.7 - 57.7) | 26.6 | 1.9 | 1.7 | (1.7 - 1.8) |
| Gastroenteritis | 60 121 | 7.8 | (7.8 - 7.9) | 2723 | 12.2 | (11.7 - 12.7) | 4.4 | 1.6 | 1.4 | (1.3 - 1.5) |
| Asthma | 56 021 | 7.3 | (7.2 - 7.4) | 2402 | 10.8 | (10.3 - 11.2) | 3.5 | 1.5 | 1.4 | (1.4 - 1.5) |
| Dental | 28 771 | 3.7 | (3.7 - 3.8) | 2091 | 9.4 | (9.0 - 9.8) | 5.7 | 2.5 | 2.6 | (2.5 - 2.7) |
| Acute upper respiratory infections^b^ | 35 428 | 4.6 | (4.6 - 4.7) | 2081 | 9.3 | (8.9 - 9.7) | 4.7 | 2.0 | 1.8 | (1.8 - 1.9) |
| Skin infection | 12 058 | 1.6 | (1.5 - 1.6) | 1291 | 5.8 | (5.5 - 6.1) | 4.2 | 3.6 | 3.6 | (3.4 - 3.8) |
| Bacterial/unspecified pneumonia | 20 803 | 2.7 | (2.7 - 2.7) | 1212 | 5.4 | (5.1 - 5.7) | 2.7 | 2.0 | 1.9 | (1.8 - 2.0) |
| Otitis media | 6668 | 0.9 | (0.8 - 0.9) | 379 | 1.7 | (1.5 - 1.9) | 0.8 | 1.9 | 1.8 | (1.6 - 2.0) |
| Vaccine preventable diseases | 1476 | 0.2 | (0.2 - 0.2) | 133 | 0.6 | (0.5 - 0.7) | 0.4 | 3.0 | 2.8 | (2.3 - 3.4) |
| Dermatitis/eczema | 2478 | 0.3 | (0.3 - 0.3) | 131 | 0.6 | (0.5 - 0.7) | 0.3 | 2.0 | 1.8 | (1.5 - 2.1) |
| Gastro oesophageal reflux | 3491 | 0.5 | (0.4 - 0.5) | 112 | 0.5 | (0.4 - 0.6) | <0.1 | 1.0 | 0.9 | (0.7 - 1.1) |
| Constipation | 2922 | 0.4 | (0.4 - 0.4) | 105 | 0.5 | (0.4 - 0.6) | 0.1 | 1.3 | 1.2 | (1.0 - 1.5) |
| Urinary tract infection (>=5 yrs) | 1303 | 0.2 | (0.2 - 0.2) | 54 | 0.2 | (0.2 - 0.3) | <0.1 | 1.0 | 1.2 | (0.9 - 1.5) |
| Nutritional deficiency | 411 | 0.1 | (<0.1 - 0.1) | 31 | 0.1 | (0.1 - 0.2) | <0.1 | 1.0 | - | - |
| Acute rheumatic fever | 18 | <0.1 | (<0.1 - <0.1) | 12 | 0.1 | (<0.1 - 0.1) | 0.1 | - | - | - |
| Bronchiectasis | 49 | <0.1 | (<0.1 - <0.1) | 5 | <0.1 | (<0.1 - 0.1) | <0.1 | - | - | - |
| Chronic rheumatic heart disease | <5 | <0.1 | (<0.1 - <0.1) | <5 | <0.1 | (<0.1 - <0.1) | <0.1 | - | - | - |
| *Other avoidable hospitalisations* | - | - | - | - | - | - | - | - | - | - |
| Acute bronchiolitis | 50 253 | 6.5 | (6.5 - 6.6) | 4525 | 20.3 | (19.7 - 20.9) | 13.8 | 3.1 | 2.3 | (2.2 - 2.4) |
| Viral infection of unspecified site | 28 883 | 3.8 | (3.7 - 3.8) | 1185 | 5.3 | (5.0 - 5.6) | 1.5 | 1.4 | 1.3 | (1.2 - 1.4) |
| Croup, acute laryngitis, tracheitis | 17 375 | 2.3 | (2.2 - 2.3) | 792 | 3.5 | (3.3 - 3.8) | 1.2 | 1.5 | 1.4 | (1.3 - 1.5) |
| Febrile convulsions | 10 505 | 1.4 | (1.3 - 1.4) | 518 | 2.3 | (2.1 - 2.5) | 0.9 | 1.6 | 1.6 | (1.4 - 1.7) |
| Viral pneumonia | 3377 | 0.4 | (0.4 - 0.5) | 154 | 0.7 | (0.6 - 0.8) | 0.3 | 1.8 | 1.5 | (1.2 - 1.7) |
| Viral/other/unspecified meningitis | 1457 | 0.2 | (0.2 - 0.2) | 77 | 0.3 | (0.3 - 0.4) | 0.1 | 1.5 | 1.7 | (1.4 - 2.2) |
| Osteomyelitis | 731 | 0.1 | (0.1 - 0.1) | 37 | 0.2 | (0.1 - 0.2) | 0.1 | 2.0 | - | - |
| Meningococcal disease | 411 | 0.1 | (<0.1 - 0.1) | 35 | 0.2 | (0.1 - 0.2) | 0.1 | 2.0 | - | - |
| Bacterial meningitis | 243 | <0.1 | (<0.1 - <0.1) | 20 | 0.1 | (0.1 - 0.1) | 0.1 | - | - | - |
| Tuberculosis | 18 | <0.1 | (<0.1 - <0.1) | 5 | <0.1 | (<0.1 - 0.1) | <0.1 | - | - | - |

eTable 2 *continued*.

|  | **Non-Aboriginal**  (Total person-years follow-up = 7 681 406) | | | **Aboriginal**  (Total person-years follow-up = 223 190) | | | **RD** | **ARR** | **aARR^c^ (95% CI)** | |
| --- | --- | --- | --- | --- | --- | --- | --- | --- | --- | --- |
|  | **Admissions**  **(N)** | **Admission Rate (AR)^a^** | **95% CI** | **Admissions**  **(N)** | **AR ^a^** | **95% CI** |  |  |  |  |
| **Non-avoidable hospitalisations** | 73 914 | 9.6 | (9.6 - 9.7) | 2245 | 10.1 | (9.6 - 10.5) | 0.5 | 1.1 | 0.9 | (0.8 - 1.0) |
| Urinary tract infection  (<5 years of age) | 12 633 | 1.6 | (1.6 - 1.7) | 470 | 2.1 | (1.9 - 2.3) | 0.5 | 1.3 | 1.2 | (1.1 - 1.3) |
| Epilepsy/status epilepticus | 7295 | 0.9 | (0.9 - 1.0) | 450 | 2.0 | (1.8 - 2.2) | 1.1 | 2.2 | 1.8 | (1.5 - 2.1) |
| Neoplasm (malignant or not) | 21 649 | 2.8 | (2.8 - 2.9) | 369 | 1.7 | (1.5 - 1.8) | -1.1 | 0.6 | 0.6 | (0.5 - 0.7) |
| Inguinal hernia | 10 692 | 1.4 | (1.4 - 1.4) | 364 | 1.6 | (1.5 - 1.8) | 0.2 | 1.1 | 0.9 | (0.8 - 1.0) |
| Abdominal/pelvic pain | 6075 | 0.8 | (0.8 - 0.8) | 205 | 0.9 | (0.8 - 1.1) | 0.1 | 1.1 | 1.2 | (1.1 - 1.4) |
| Acute appendicitis | 3258 | 0.4 | (0.4 - 0.4) | 106 | 0.5 | (0.4 - 0.6) | 0.1 | 1.3 | 1.3 | (1.1 - 1.6) |
| Coagulation defects | 2321 | 0.3 | (0.3 - 0.3) | 85 | 0.4 | (0.3 - 0.5) | 0.1 | 1.3 | 0.9 | (0.6 - 1.3) |
| Other avoidable | 2642 | 0.3 | (0.3 - 0.4) | 78 | 0.3 | (0.3 - 0.4) | <0.1 | 1.0 | 0.8 | (0.4 - 1.6) |
| Type 1 diabetes | 2693 | 0.4 | (0.3 - 0.4) | 72 | 0.3 | (0.3 - 0.4) | -0.1 | 0.8 | 0.9 | (0.7 - 1.2) |
| Cystic fibrosis | 1344 | 0.2 | (0.2 - 0.2) | 30 | 0.1 | (0.1 - 0.2) | -0.1 | 0.5 | - | - |
| Immune disorders | 3312 | 0.4 | (0.4 - 0.4) | 16 | 0.1 | (<0.1 - 0.1) | -0.3 | 0.3 | - | - |

AR, admission rate; CI, confidence interval; RD, rate difference; ARR, Admission Rate Ratio; aARR, Adjusted Admission Rate Ratio. a. Per 1000 person-years. b. excluding croup. c. Adjusted for age and sex. Number of admissions suppressed if less than 5 admissions recorded during the study period. Adjusted ARR not calculated if less than 50 hospitalisations recorded for Aboriginal or non-Aboriginal children during the study period.
